# Temperature-guided high and very high-power short duration ablation for atrial fibrillation treatment: the peQasus multicentre study

**DOI:** 10.1093/europace/euae284

**Published:** 2024-11-07

**Authors:** Christian-Hendrik Heeger, Alexandre Almorad, Daniel Scherr, Nándor Szegedi, Sebastian Seidl, Jakub Baran, Mattias Duytschaever, Dhiraj Gupta, Dominik Linz, Evgeny Lyan, Domenico Della Rocca, László Gellér, Sebastien Knecht, Peter Calvert, Samuel Meilak, Georgios Leventopoulos, Sorin Stefan Popescu, Martin Rauber, Georgios Kollias, Michał Niedzwiedz, Andrea Sarkozy, Marc Badoz, Martin Manninger-Wünscher, Vanessa Sciacca, Christian Sohns, Matthew R Ginks, Helmut Pürerfellner, Roland R Tilz

**Affiliations:** Department of Rhythmology, Cardiology and Internal Medicine, Asklepios Klinik Hamburg Altona, Paul Ehrlich Straße 1, 22763 Hamburg, Germany; University Heart Center Lübeck, Department of Rhythmology, University Hospital Schleswig-Holstein, Ratzeburger Allee 160, 23562 Lübeck, Germany; German Center for Cardiovascular Research, Partner Site Hamburg/Kiel/Luebeck, Luebeck, Germany; Heart Rhythm Management Centre, Postgraduate Program in Cardiac Electrophysiology and Pacing, Universitair Ziekenhuis Brussel—Vrije Universiteit Brussel, Brussels, Belgium; European Reference Networks Guard-Heart, Laarbeeklaan 101, 1090 Brussels, Belgium; Department of Cardiology, Universität Graz, Graz, Austria; Heart and Vascular Center, Semmelweis University, Budapest, Hungary; Ordensklinikum Linz Elisabethinen, Linz, Austria; Department of Internal Medicine and Cardiology University Clinical Center, Medical University of Warsaw, Warsaw, Poland; Division of Clinical Electrophysiology, Department of Cardiology, Centre of Postgraduate Medical Education, Grochowski Hospital, Warsaw, Poland; Department of Cardiology, AZ Sint-Jan Hospital, Bruges, Belgium; Liverpool Heart and Chest Hospital, Faculty of Health and Life Sciences, University of Liverpool, Liverpool, UK; Department of Cardiology, Maastricht University Medical Center, Maastricht, The Netherlands; University Heart Center Kiel, University Hospital Schleswig-Holstein, Kiel, Germany; Heart Rhythm Management Centre, Postgraduate Program in Cardiac Electrophysiology and Pacing, Universitair Ziekenhuis Brussel—Vrije Universiteit Brussel, Brussels, Belgium; Heart and Vascular Center, Semmelweis University, Budapest, Hungary; Department of Cardiology, AZ Sint-Jan Hospital, Bruges, Belgium; Liverpool Heart and Chest Hospital, Faculty of Health and Life Sciences, University of Liverpool, Liverpool, UK; Oxford University Hospitals NHS Foundation Trust, Oxford, UK; John Radcliffe Hospital, Oxford, UK; Department of Cardiology, University Hospital of Patras, Patras, Greece; University Heart Center Lübeck, Department of Rhythmology, University Hospital Schleswig-Holstein, Ratzeburger Allee 160, 23562 Lübeck, Germany; Department of Cardiology, Universität Graz, Graz, Austria; Department of Cardiology, Medical University of Ljubljana, Ljubljana, Slovenia; Ordensklinikum Linz Elisabethinen, Abteilung für Innere Medizin 2 - Kardiologie, Angiologie und Intensivmedizin, Linz, Austria; Division of Clinical Electrophysiology, Department of Cardiology, Centre of Postgraduate Medical Education, Grochowski Hospital, Warsaw, Poland; Heart Rhythm Management Centre, Postgraduate Program in Cardiac Electrophysiology and Pacing, Universitair Ziekenhuis Brussel—Vrije Universiteit Brussel, Brussels, Belgium; European Reference Networks Guard-Heart, Laarbeeklaan 101, 1090 Brussels, Belgium; Department of Cardiology, University Hospital of Besançon, Besançon, France; Department of Cardiology, Universität Graz, Graz, Austria; Herz- und Diabeteszentrum NRW, Universitätsklinik der Ruhr-Universität Bochum, Bad Oeynhausen, Germany; Herz- und Diabeteszentrum NRW, Universitätsklinik der Ruhr-Universität Bochum, Bad Oeynhausen, Germany; Department of Cardiology, Oxford University Hospitals NHS Foundation Trust, Oxford, UK; Ordensklinikum Linz Elisabethinen, Abteilung für Innere Medizin 2 - Kardiologie, Angiologie und Intensivmedizin, Linz, Austria; University Heart Center Lübeck, Department of Rhythmology, University Hospital Schleswig-Holstein, Ratzeburger Allee 160, 23562 Lübeck, Germany; German Center for Cardiovascular Research, Partner Site Hamburg/Kiel/Luebeck, Luebeck, Germany

**Keywords:** Atrial fibrillation, High-power short duration, Pulmonary vein isolation, Radiofrequency, Acute efficacy

## Abstract

**Aims:**

Temperature-controlled high-power short-duration (HPSD) radiofrequency catheter ablation for pulmonary vein isolation (PVI) utilizing a novel ablation catheter (QDOT Micro) with real-time assessment of catheter tip temperature aims for safer, more effective, and faster procedures.

**Methods and results:**

The peQasus study is a large European multicentre study set up to assess safety, acute efficacy, and outcomes of temperature-controlled HPSD-based PVI. The primary endpoints were safety, efficacy, and 12-month freedom from atrial tachyarrhythmias. Additionally, two strategies namely very HPSD (90 W for 4 s) only and a hybrid approach (HPSD with maximum of 50 W and vHPSD) were compared. A total of 1023 AF patients in 15 centres from nine European countries received PVI with the QDOT. Complete PVI was successfully achieved in all patients. In 699/1023 (68.3%), the vHPSD-only approach (vHPSD group) and in 324/ (31.7%) patients, the hybrid approach (hybrid group) was utilized. The mean procedure duration was 98.4 ± 37.4 min (vHPSD: 88.2 ± 34.9 min, hybrid: 117.4 ± 32.7 min, *P* < 0.001). The first-pass isolation rate of all PVs was 64% (vHPSD: 62.6%, hybrid: 67.1%, *P* = 0.187). Severe adverse events were observed in 1.7% (vHPSD: 1.6%, hybrid: 1.9%, *P* = 0.746). Twelve-month arrhythmia-recurrence-free survival was 77.1% (vHPSD: 76.8%, hybrid: 77.8%, *P* = 0.241).

**Conclusion:**

In this large multicentre study, temperature-controlled HPSD and vHPSD ablation via a novel ablation catheter provides safe and effective PVI with a relatively short procedure duration. Despite a shorter procedure time, no differences in terms of safety and freedom from arrhythmia recurrence were found irrespective of utilizing vHPSD or the hybrid approach.

## Graphical Abstract

**Graphical Abstract:** Safety, efficacy, and outcomes of temperature-guided (very) high-power short duration ablation for atrial fibrillation—the peQasus multicentre study

What’s new?

- The peQasus study aims to assess safety, efficacy, and follow-up for pulmonary vein isolation (PVI) utilizing the novel QDOT Micro ablation catheter in a large multicentre European cooperation.
- All PVs could be isolated utilizing the QDOT Micro ablation catheter.
- Radiofrequency time, procedure time, and LA dwell time were significantly shorter utilizing the vHPSD-only approach and in terms of first-pass isolation rate, no differences were found between the groups.
- The rate of periprocedural complications was low and no differences were observed between the groups, and the long-term outcome was promising and similar between the groups.

## Introduction

Catheter ablation for treatment of paroxysmal (PAF) and persistent atrial fibrillation (PersAF) by pulmonary vein isolation (PVI) has demonstrated high procedural success and promising long-term outcomes.^1^ The introduction of novel single-shot systems has yielded excellent acute and long-term success rates while reducing procedure time compared to radiofrequency (RF)-based three-dimensional mapping and point-by-point PVI.^2^

Recent advancements in single-tip RF-based PVI have been made through the implementation of contact force (CF) and ablation index (AI)-guided ablation, which shortens procedure time and enhances safety and outcomes.^3,4^ Additionally, high-power short-duration (HPSD) protocols, utilizing a maximum of 50 W, and very high-power short-duration (vHPSD) protocols with a maximum of 90 W, have been evaluated and found to significantly reduce procedure duration.^5,6^ The novel QDOT Micro ablation catheter (Biosense Webster, Inc., Diamond Bar, CA, USA) has been developed allowing for real-time assessment of catheter-to-tissue interface temperature and therefore allows temperature-controlled ablation with higher power settings compared to standard power-controlled ablation.^7^ This strategy produces significantly shallower, larger diameter lesions than standard power-controlled ablation in a very short time aiming to minimize conductive heating and increase resistive heating.^7^ However, greater thermal latency for 90 W/4 s applications has been shown in animal preparations suggesting that a significant portion of lesion is created after RF termination due to conductive tissue heating. Additionally, safety may be enhanced by potentially reducing collateral tissue damage.^8^ Two ablation modes are available. In the vHPSD mode (QMODE+), only 90 W/4 s is performed while in the QMODE, temperature-controlled AI-guided ablation with a maximum of 50 W is performed. Some operators advocate for a vHPSD-only approach, whereas others have evaluated a hybrid strategy that involves switching between both ablation modes depending on the perceived thickness of the underlying tissue (anterior: QMODE, posterior: QMODE+). Although recent data indicate promising results, the reported patient numbers remain relatively low, and there is a lack of multicentre assessments and larger patient populations.^9,10^ With every novel ablation system, efficacy and safety are crucial factors determining success or failure. Therefore, we aimed to compile periprocedural data from various European centres to enhance the quality and quantity of data regarding this innovative ablation system, with a focus on safety, efficacy, and 12-month outcomes.

## Methods

### Study population

**Figure 1:**
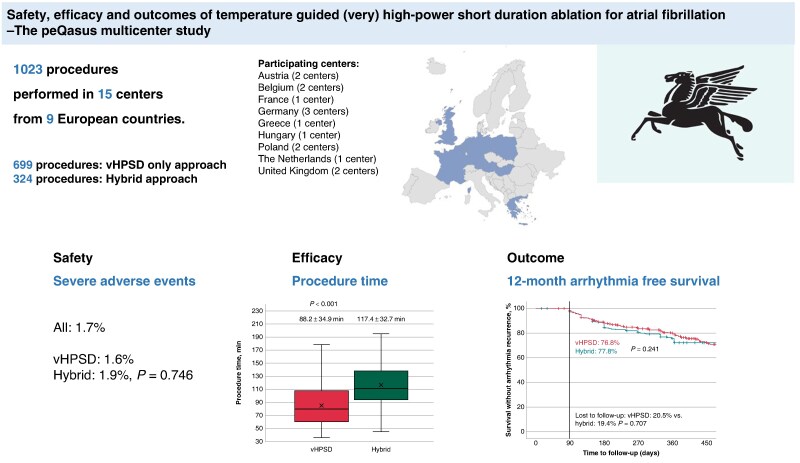
peQasus study. Overview and main findings: peQasus multicentre study.

Between July 2020 and March 2023, patients with AF who were treated with the QDOT Micro ablation catheter for *de novo* PVI were included in this multicentre study. Data were collected from 15 centres across nine countries (*Figure 1*). The participating centres from Europe are mentioned in Appendix 1.

The data acquisition from 10 of the 15 centres was prospective, while five centres provided retrospectively acquired data. The study adheres to the Declaration of Helsinki and was approved by the local institutional ethics committees. All patients provided written informed consent, and all patient information was anonymized. The peQasus multicentre study (ClinicalTrials.gov ID: NCT05710822) received approval from the local ethical review board of the University of Lübeck, Germany (AZ 15-347). Each participating centre was responsible for obtaining its own ethics approval from the local ethics committee.

### Aim of the study

The aim of the study was to assess safety, acute efficacy, and 12-month follow-up after *de novo* PVI utilizing the QDOT Micro ablation catheter. Additionally, two subgroups were designed in terms of utilizing vHPSD (90 W/4 s, QMODE+) only (vHPSD group) or a hybrid approach (either utilizing temperature-controlled AI-guided catheter ablation with a maximum of 50 W (QMODE) or a mixture of QMODE and vHPSD (QMODE+) (hybrid group). Both approaches have been recently evaluated and were found to be safe and effective.^11,12^

For this purpose, procedural efficacy and periprocedural data were assessed and analysed. Furthermore, the incidence of periprocedural complications, such as bleeding events (defined as bleedings requiring medical action), pericardial effusion and/or pericardial tamponade, cerebral stroke or clinical apparent oesophageal injuries. Periprocedural complications were defined according to latest guidelines.^1^ Only adverse events adjudicated as possible, probable, or definitely related to the ablation procedure were mentioned as safety events. An adverse event was considered serious if it resulted in permanent injury or death, required an intervention for treatment, or required hospitalization for more than 24 h. Additionally, long-term follow-up was assessed and analysed. Recurrence of AF or atrial tachycardia (AT) was defined as freedom from documented AF/AT recurrence 12 months after PVI, including a 90-day blanking period. Recurrence was defined as any ECG-documented atrial tachyarrhythmia lasting for at least 30 s, including AF, AT, and atrial flutter. Patients completed outpatient clinic visits at 3, 6, and 12 months, including ECGs and 24 h-Holter ECGs.

### Intraprocedural management

The detailed intraprocedural management for 3D mapping, AI-guided as well as vHPSD-based PVI has been described in previous studies from different groups.^11–14^ In brief, the procedures were performed under deep sedation with propofol, consciousness sedation with opiates, or full general anaesthesia with ventilation depending on the individual centres approach. Two to three right femoral vein punctures (ultrasound guided in 14/15 centres (93%) were performed and short sheaths were inserted. Prior to transseptal puncture (TSP), a diagnostic catheter was introduced and positioned inside the coronary sinus. Single or double TSP was performed under fluoroscopic guidance using a modified Brockenbrough technique with 8.5 F transseptal sheaths and puncture needle (SL1 or SL0 sheath and BRK-1 TSP needle, St. Jude Medical, Inc., St. Paul, MN, USA). Pulmonary vein (PV) angiography (3/15, 20% centres), or preprocedural left atrial CT angiography (3/15, 20% centres), was performed to assess the PV anatomy in some centres. The sheaths were continuously flushed with heparinized saline (10 mL/h). After or directly before TSP, heparin boluses were administered targeting an activated clotting time of >300 s.

### Ablation procedure

An oesophageal temperature probe (CIRCA S-CATH, Circa Scientific, Englewood, USA) or SensiTherm (Abbott) was advanced into the oesophagus to monitor the oesophageal temperature (Teso) in some cases, based on the individual centres approach. Three-dimensional electroanatomic LA reconstruction (CARTO 3, Biosense Webster) was performed either via fast anatomical mapping either with a multi-electrode mapping catheter (Octaray, Pentaray or Lasso Nav, Biosense Webster) or the ablation catheter. For the LA voltage map, the bipolar voltage reference interval was set according to each centre preferences: 0.05–0.5 mV (4/15, 27% centres), 0.1–0.5 mV (3/15, 20% centres), 0.2–0.5 mV (6/15, 40% centres), 0.1–0.3 mV (1/15, 7% centres), and 0.3–0.6 mV (1/15, 7% centres). The ipsilateral PVs were tagged according to 3D mapping. During the procedures, special attention was drawn for audible steam pops and all catheters were checked for charring after removal. After PVI, a multi-electrode mapping catheter or the ablation catheter was positioned inside the ipsilateral PVs to verify durable PVI. In case of previously known or periprocedural typical atrial flutter, cavotricuspid isthmus (CTI) ablation was performed in both groups. Additional ablation strategies have been performed according to the individual centres’ strategies. The presence or absence of first-pass isolation of the ipsilateral PVs was evaluated as described previously.^14,15^

### vHPSD-only approach

**Figure 2:**
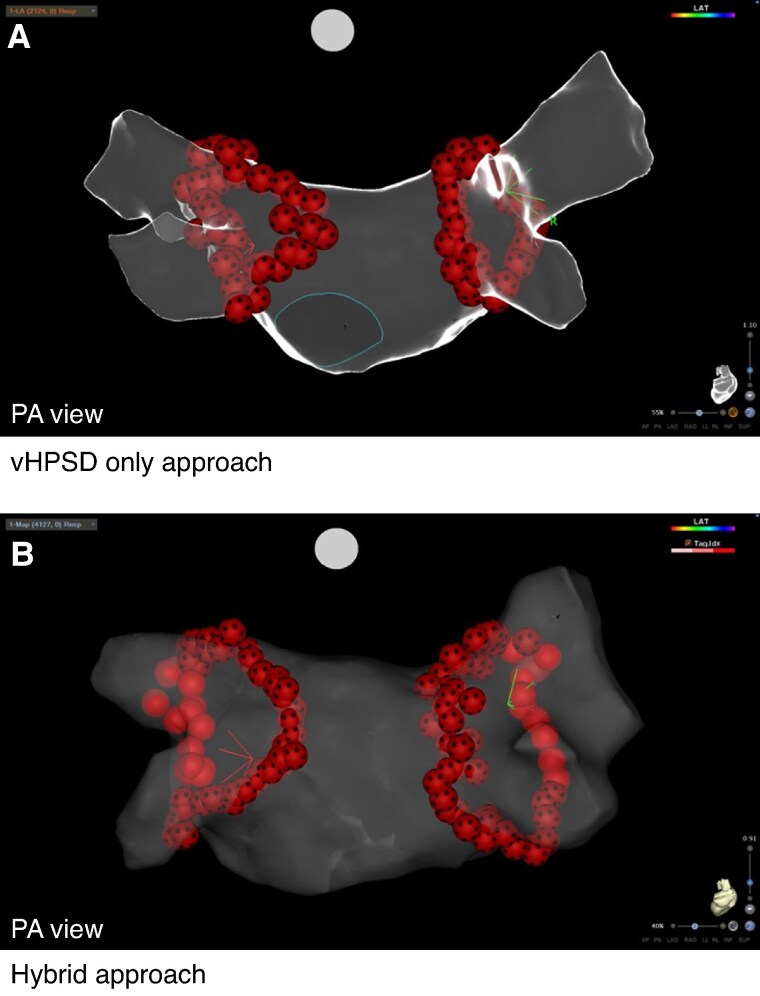
Final lesions set after vHPSD-only (*A*) and hybrid (*B*) approaches. Three-dimensional electroanatomical reconstruction (CARTO 3, UNIVIEW module, Biosense Webster) of the left atrium in posterior anterior (left) view. (*A*) Please note the two circles of very high-power short duration applications by 90 W/4 s (QMODE+ mode, red–black tags) utilizing a very high-power short duration only approach. (*B*) Please note the two circles of the hybrid approach utilizing very high-power short duration applications by 90 W/4 s (QMODE+ mode, red–black tags) posterior and high-power short duration applications by 50 W (QMODE, ablation index-guided applications, red tags) anterior encircling the right and left pulmonary veins.

In the vHPSD group, all applications were performed with vHPSD applications (90 W, 4 s; QMODE+). The target temperature of the temperature-controlled ablation was 60°C based on the hottest surface thermocouple.^7^ The irrigation flow rate delays the energy application for a minimum of 2 s before and 4 s after each RF application. For all applications utilizing a vHPSD approach, a precise focusing on the measurement of the inter-lesion distance (intertag-distance 3–4 mm) was performed.^11,12,14,15^ The target CF range was 10–25 g. The final lesion set after vHPSD-based PVI is shown in *Figure 2A*. In case of no first-pass isolation, touch-up applications were performed with vHPSD in 11/15 centres, while 4/15 centres switched to QMODE.

### Hybrid approach

In the hybrid group, vHPSD (QMODE+) and/or temperature-controlled AI-guided ablation (QMODE) were used. As described above for the posterior aspect, vHPSD applications with an inter-lesion distance of 3–4 mm was performed while for the anterior aspect, QMODE applications were used. Energy application in QMODE was set at 50 W. Target range for CF was 10–25 g. Target AI was 500–550 or 380–400 for the anterior and posterior segments, respectively.^13^ The inter-lesion distance was set to 5–6 mm. In case of no first-pass isolation, 14/15 centres stayed on 50 W while 1/15 centres switched to 40 W. The final lesion set after hybrid approach based PVI is shown in *Figure 2B*.

### Post-procedural care

After sheath removal, a figure-of-eight suture and/or a pressure bandage were used to prevent femoral bleeding. The pressure bandage was removed after 4–8 h, and the figure-of-eight suture was removed on the next day. Following ablation, patients underwent transthoracic echocardiography as per institutional standard to rule out a pericardial effusion. Direct oral anticoagulants were typically re-initiated 6 h post-ablation. Anticoagulation was continued for at least 2 months (2 months: six centres, 40%; 3 months: eight centres, 53%; 6 months: one centre, 7%) and continued thereafter based on the patients individual CHA_2_DS_2_-VASc score. Antiarrhythmic drugs or a new antiarrhythmic drug was prescribed as per institutional standard and was discontinued latest 2 months post-ablation. Proton-pump inhibitors were administered as per institutional standard. Following a 3-month blanking period, patients completed outpatient clinic visits, including ECG and 24–72 h-Holter ECG at 3, 6, and 12 months. Additional outpatient clinic visits and/or ECG monitoring were arrangements in cases of symptoms suggestive of arrhythmia recurrence.

### Statistical analysis

All analyses were performed using STATA software version 14.0 (STATA Corp, Lake Drive Way, TX, USA). Distributions of continuous variables were tested for normality using the Shapiro–Wilk test. Continuous variables are expressed as mean ± standard deviation (SD) for normally distributed, or as median [inter-quartile range (IQR)], for non-normally distributed otherwise. Categorical variables are reported as counts (percentage). Comparisons of continuous variables were performed using the Student’s *t*-test for two groups or ANOVAs in case of multiple groups, or the corresponding non-parametric test, if not normally distributed. Comparisons of categorical variables were performed using χ^2^ or Fisher’s exact test, as appropriate. Recurrence-free survival was estimated with the Kaplan–Meier method. All *P*-values reported are two-sided, and a *P*-value of <0.05 was considered statistically significant.

## Results

### Patient characteristics

A total of 1023 patients (64.9% paroxysmal AF) were enrolled from 15 centres from nine European countries. Complete PVI of all pulmonary veins was successfully achieved in 100% of patients. Patient baseline characteristics are shown in *Table 1*.

**Table 1:** Baseline patient characteristics

Variable	All	vHPSD	Hybrid	*P*
Patients	1023	699	324	
Age, years	63.2 ± 10.1	63.1 ± 10.4	63.5 ± 9.3	0.299
BMI	28.0 ± 4.9	27.8 ± 5.0	28.3 ± 4.7	0.353
LA volume, mL/m^2a^	40.4 ± 12.9	40.1 ± 12.4	41.6 ± 14.4	0.356
Duration of AF, months	29.3 ± 44.5	30 ± 45.7	28.7 ± 45.7	0.221
Female sex	339 (31)	252 (36)	87 (26.9)	0.004
Paroxysmal AF	666 (65.1)	448 (64.1)	218 (67.3)	0.319
Congestive heart failure	99 (9.7)	56 (8)	43 (13.3)	0.008
Arterial hypertension	504 (49.3)	388 (55.5)	116 (35.8)	<0.001
Diabetes mellitus type 2	122 (11.9)	91 (13)	31 (9.6)	0.113
Coronary artery disease	135 (13.2)	92 (13.2)	43 (13.3)	0.964
Previous TIA/stroke	61 (6)	45 (6.4)	16 (4.9)	0.346
CHA_2_DS_2_-VASc score	1.9 ± 1.5	2.0 ± 1.5	1.7 ± 1.4	0.221

Values are counts, *n* (%) or mean (±SD).

AF, atrial fibrillation; BMI, body mass index; LA, left atrium.

^a^Per body surface area.

### Procedural characteristics

**Figure 3:**
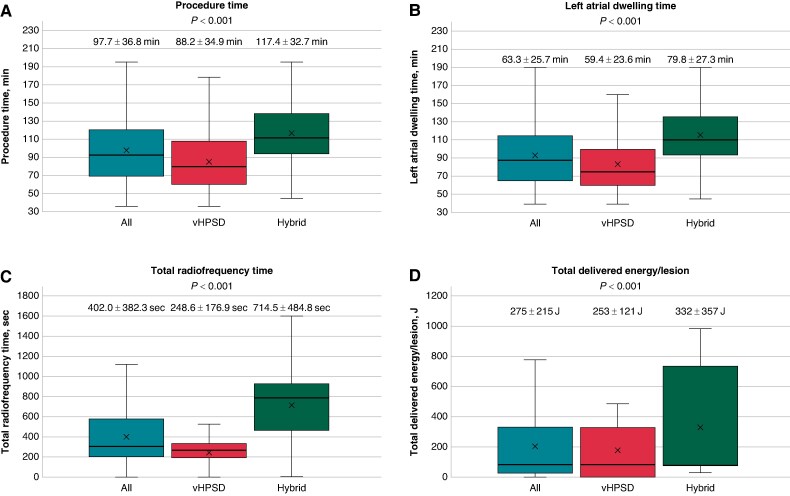
Periprocedural data: (*A*) procedure time, (*B*) left atrial dwelling time, (*C*) total radiofrequency time, (*D*) delivered energy/lesion.

Procedural characteristics are depicted in *Tables 2* and *3*. The distribution of procedure time and rate of first-pass isolation for all centres is shown in Supplementary material online, *Figure 1A* and *B*. The procedures were performed under deep sedation (6/15, 40% of centres), conscious sedation (2/15, 13.3% of centres), or general anaesthesia (7/15, 46.7% centres) depending on the individual centres approach. All pulmonary veins have been isolated utilizing the QDOT Micro ablation catheter. In 742/1023 (72.5%) patients, only PVI was performed, while the remaining patients received additional ablation (cavotricuspid isthmus block, additional atrial lesion sets). A vHPSD-only approach (vHPSD group) utilizing the QMODE+ was performed in 699/1023 (68.3%) patients, and a hybrid approach (hybrid group) was used in 324/1023 (31.7%) patients. Six centres (40%) used the vHPSD approach, four centres (27%) the hybrid approach, and five centres (33%) utilized both approaches. The mean procedure duration was 98.4 ± 37.4 min. Significantly shorter procedure times (vHPSD: 88.2 ± 34.9 min, hybrid: 118.6 ± 33.6 min, *P* < 0.001), LA dwell times (vHPSD: 59.4 ± 23.6 min, hybrid: 79.8 ± 27.3 min, *P* < 0.001), and radiofrequency times (vHPSD: 248.6 ± 176.9 s, hybrid: 714.5 ± 484.8 s, *P* < 0.001), were observed for the vHPSD group (Figure *3A–C*).

**Table 2:** Procedural details

Variable	All	vHPSD	Hybrid	*P*
Number of patients	1023	699	324	
Number of PVs	4092	695	320	
% of isolated PVs	100	100	100	0.999
FAAVI	607/949 (64)	419/669 (62.6)	188/280 (67.1)	0.187
Total procedure time, min	97.7 ± 36.8	88.2 ± 34.9	117.4 ± 32.7	<0.001
Total procedure time, min (PVI only)	92.8 ± 34.3	83.6 ± 31.2	115.2 ± 31.2	<0.001
Total LA dwelling time, min	63.3 ± 25.7	59.4 ± 23.6	79.8 ± 27.3	<0.001
Total fluoroscopy time, min	9.1 ± 18.2	9.9 ± 22.1	7.7 ± 7.2	<0.001
Total radiofrequency time, s	402 ± 382	248.6 ± 176.9	714.5 ± 484.8	<0.001
Total number of applications	76.2 ± 23.9	79.4 ± 20.9	68.7 ± 28.4	<0.001
Mean application duration, s	4.8 ± 2.8	4.0 ± 0.2	11.3 ± 4.9	<0.001
Mean contact force, g	15.4 ± 3.6	15.1 ± 3.5	16.5 ± 3.8	0.242
Mean power/application, Watt	83.7 ± 12.6	88.9 ± 5.1	66.4 ± 14.8	<0.001
Total delivered energy/lesion, joule	275 ± 215	253 ± 121	332 ± 357	<0.001
PVI only	742 (72.5)	532 (76.1)	210 (64.8)	<0.001
Additional CTI block	265 (26)	158 (23)	107 (33)	<0.001
Additional LA ablation	29 (2.8)	22 (3.1)	7 (2.2)	0.376
General anaesthesia	365	144 (20.6)	221 (68.2)	<0.001

Values are counts, *n* (%) or mean (±SD).

PV(s), pulmonary vein(s); PVI, pulmonary vein isolation; FAAVI, first attempt all veins isolated; LA, left atrium; min, minutes; s, seconds; g, gram.

**Table 3:** Procedural details—individual pulmonary vein

Variable	All	vHPSD	Hybrid	*P*
Right-sided PVs	1023	699	324	
% of isolated PVs	100	100	100	0.999
Total ablation time, s	265.0 ± 171.2	168.6 ± 94.0	428.3 ± 147.0	<0.001
Total number of applications	38.3 ± 12.0	40.6 ± 12.3	34.4 ± 10.4	0.002
Mean application duration, s	4.5 ± 2.2	4 ± 0.2	10.3 ± 4.3	<0.001
Mean contact force, g	15.4 ± 3.9	15.1 ± 3.4	17.1 ± 5.4	0.492
Mean power/application, Watt	85.1 ± 11.6	89.4 ± 4.2	66.8 ± 15.1	<0.001
FAVI	703/949 (74.1)	492/669 (73.5)	211/280 (75.4)	0.561
Left-sided PVs	1023	699	324	
% of isolated PVs	100	100	100	0.999
Total ablation time, s	268.1 ± 190.7	153.0 ± 57.8	460.9 ± 179.7	<0.001
Total number of applications	36.3 ± 11.3	38.0 ± 11.7	33.4 ± 11.7	<0.001
Mean application duration, s	4.6 ± 2.3	4.0 ± 0.3	11.0 ± 4.3	<0.001
Mean contact force, g	13.4 ± 3.3	13.2 ± 3.1	14.4 ± 3.8	0.214
Mean power/application, Watt	84.7 ± 12.4	89.1 ± 6.3	66.0 ± 14.7	<0.001
FAVI	740/948 (78.1)	516/667 (77.4)	224/281 (79.7)	0.424

Values are counts, *n* (%) or mean (±SD). FAVI: first attempt veins isolated = first-pass isolation.

PV(s), pulmonary vein(s); FAVI, first attempt vein isolated; s, seconds; g, grams.

For an analysis of PVI-only procedures, all patients with additional ablation strategies (*n* = 281) were excluded. For PVI-only procedures, the procedures times were: 92.8 ± 34.3 min, vHPSD: 83.6 ± 31.2 min, and hybrid: 115.2 ± 31.2 min (*P* < 0.001).

The total number of applications was lower (vHPSD: 79.4 ± 20.9, hybrid: 68.7 ± 28.4, *P* < 0.001) while the total delivered energy/lesion (vHPSD: 253J ± 121J vs. hybrid: 332J ± 357J, *P* < 0.001) was higher in the hybrid group (*Figure 3D*).

The overall first-pass isolation rate was 64%, with no difference between vHPSD (62.6%), and hybrid (67.1%), *P* = 0.187. For right-sided PVs, the rate of first-pass isolation was 74.1% (vHPSD: 73.5%, hybrid: 75.4%, *P* = 0.561) while for left-sided PVs, it was 78.1% (vHPSD: 77.4%, hybrid: 79.7%, *P* = 0.424).

### Safety

Severe adverse events were seen in *n* (1.7%) patients with no differences between the groups (1.7% vHPSD vs. 1.9% hybrid, *P* = 0.746), *Table 4*. Cardiac tamponade requiring percutaneous drainage was observed in two patients of the vHP group (0.3% vs. 0%, *P* = 0.568). Both patients made good recovery without any surgical intervention. Groin bleeding requiring blood transfusion or intervention was observed in *n* (1.4%) patients (vHPSD: 1.1% vs. hybrid: 1.9%, *P* = 0.365). One patient in the vHP group experienced post-procedural pulmonary embolism. No patients experienced stroke or transient ischemic attack (TIA), phrenic nerve injury, atrioesophageal fistula, or air embolism. Minor periprocedural complications were observed in *n* (0.8%). No differences were observed between the two groups (0.7% vHP vs. 0.9% hybrid, *P* = 0.722).

**Table 4:** Periprocedural complications

Variable	All	vHPSD	Hybrid	*P*
Number of patients	1023	699	324	
Severe adverse events (%)	17 (1.7)	11 (1.6)	6 (1.9)	0.746
Cardiac tamponade (%)	2 (0.2)	2 (0.3)	0 (0)	0.568
Severe bleeding (%)	14 (1.4)	8 (1.1)	6 (1.9)	0.365
Phrenic nerve injury	0	0	0	0.999
Atrioesophageal fistula	0	0	0	0.999
Stroke or TIA	0	0	0	0.999
Pulmonal embolism (%)	1 (0.1)	1 (0.1)	0	0.308
Minor complications (%)	8 (0.8)	5 (0.7)	3 (0.9)	0.722
Minor bleeding (%)	3 (0.3)	2 (0.3)	1 (0.3)	0.949
Pericardial effusion (%)	4 (0.4)	3 (0.4)	1 (0.3)	0.774
Air embolism	0	0	0	0.999
Clinical apparent oesophagus injury (%)	1 (0.1)	0	1 (0.3)	0.308
Oesophageal temperature probe (%)	348 (34)	191 (27.3)	157 (48.5)	<0.001
Temperature rise > 38.5°C (%)	155 (44.5)	85 (44.5)	70 (44.6)	0.898
Charring on catheter tip	0	0	0	0.999
Audible steam pop, *n* (%)	1 (0.1)	1 (0.1)	0	0.308

Values are counts, *n* (%) or mean (±SD).

One documented audible steam pop was reported for the vHPSD group. This event did not result in a periprocedural complication. No catheter tip charring was detected in any patient. An oesophageal temperature probe was utilized in 34% of patients with 27.3% for vHPSD and 48.5% of hybrid patients (*P* < 0.001). A temperature of >38.5°C was detected in 44.5% patients (vHP: 44.5% vs. hybrid: 44.6%, *P* = 0.898). Clinically apparent oesophagus injury was observed in one patient (0.1%) of the hybrid group. No long-term sequalae was reported in this patient.

#### Follow-up and clinical success

**Figure 4:**
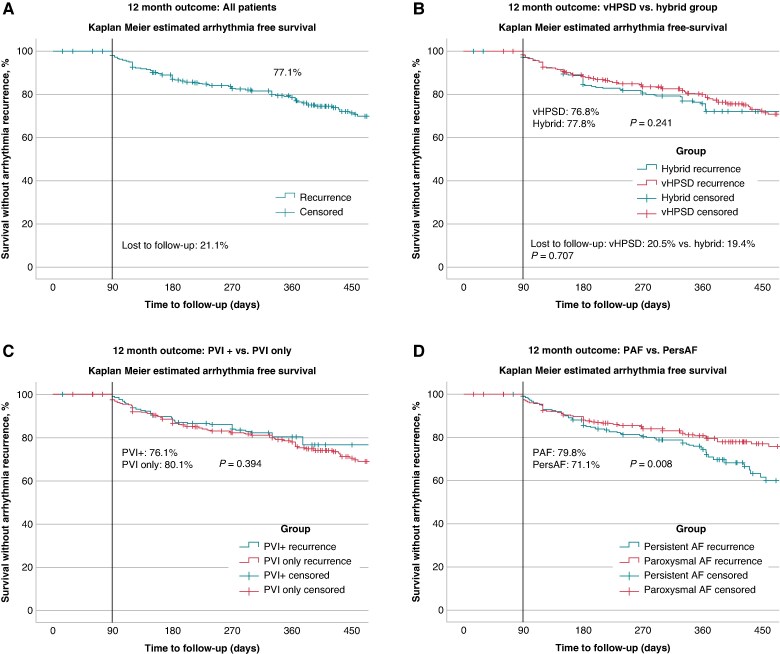
Twelve-month follow-up and findings of repeat procedures. (*A*) Kaplan–Meier Estimates with 12-month follow-up after the index PVI utilizing the QDOT Micro ablation catheter—all patients. (*B*) Kaplan–Meier Estimates with 12-month follow-up after the index PVI utilizing the QDOT Micro ablation catheter and comparison of the vHPSD strategy and hybrid approaches. No statistical differences were found concerning 12-month freedom from atrial tachyarrhythmias. (*C*) Comparison of the PVI vs. PVI+ strategies. No statistical differences were found concerning 12-month freedom from atrial tachyarrhythmias. PVI+ = PVI plus CTI and/or LA ablation. (*D*) Comparison of the patients with PAF vs. PersAF. A statistical better outcome for PAF patients has been observed.

In a total of 817/1023 patients (79.9%), 12 months follow-up was available [the rate of lost to follow-up was not different between the groups (vHPSD: 20.5%, vs. hybrid: 19.4%, *P* = 0.707)]. Three-month Holter was available in 72.3% of patients and 6-month Holter was available in 76.4% of patients while 12-month Holter was available in 78.8% of patients. The rate of 12-month AF/AT free survival after a 90-day blanking period was 77.1% (vHPSD: 76.8% vs. hybrid: 77.8%, *P* = 0.241, *Figure 4*). The mean time to recurrence was 211.8 ± 221.2 days. The median follow-up duration was 360 (360, 510) days. The comparison of follow-up of patients treated with a PVI vs. PVI plus further ablation strategies found no differences (*P* = 0.394). Concerning patients with PAF or PersAF 12-month AF/AT free survival after a 90-day blanking period was PAF: 79.8% vs. PersAF: 71.1%, respectively, *P* = 0.008.

## Discussion

The peQasus study aims to assess safety, efficacy, and follow-up for PVI utilizing the novel QDOT Micro ablation catheter in a large multicentre European cooperation. Additionally, two different ablation strategies (e.g. vHPSD-only and hybrid approach) were compared.

The major findings are as follows:

1. All PVs could be isolated utilizing the QDOT Micro ablation catheter.
2. Radiofrequency time, procedure, time and LA dwell time were significantly shorter utilizing the vHPSD-only approach.
3. In terms of first-pass isolation rate, no differences were found between the groups.
4. The rate of periprocedural complications was low, and no differences were observed between the groups.
5. The long-term outcome was promising and similar between the groups.

The advantages of single-shot devices for PVI are shorter procedure times, learning curves, and safety aspects. However, point-by-point RF ablation catheter allows for a better personalized approach for extra PV ablation while single short devices are usually only utilized for posterior wall isolation. Recently, the HPSD concept of RF-based PVI with increased power and shorter duration was introduced and efficacy and safety were shown in previous studies in a power-controlled ablation mode.^16,17^ A reduction of the procedure time has been shown for the vHPSD concept utilizing 90 W for 4 s in a temperature-controlled mode that was recently realized by the QDOT Micro catheter.^17^ The six thermocouples of the QDOT Micro allow for precise temperature measurement and power as well as irrigation flow modulation to avoid tissue overheating, collateral damage, catheter tip charring, and steam pops.^11^

With the QDOT Micro catheter, it is feasible to switch from the QMODE+ to the QMODE. Some operators prefer a QMODE+ only approach while others are performing a hybrid approach of mixing QMODE+ and QMODE. No large-scale head-to-head comparisons have been conducted to date. Here, we showed no differences in terms of safety and efficacy for both strategies.

In our study, no charring was observed and the rate of audible steam pops and clinical apparent oesophageal injuries were very low, suggesting a favourable safety profile. This was also previously evaluated by several studies.^18–20^ However, recently, a higher than expected rate of symptomatic pulmonary vein stenosis has been found for patients treated with vHPSD.^21^

In the peQasus study, no symptomatic pulmonary vein stenosis has been found.

The application duration and consequently the total RF ablation time were significantly reduced utilizing vHPSD only. This translated into significantly reduced LA dwell times and procedure times that is in line previous single-centre studies.^11,12^

The rate of first-pass isolation was within the range of existing published data and comparable between the groups.^11,12^ Notably, PVI was systematically achieved by QMODE+ applications only. For PVI-only procedures, a mean procedure time of <90 min was observed for the vHPSD strategy, which offers short procedures times comparable to single-shot devices.^22–24^ Due to the fact that lesion formation of vHPSD applications creates wider but shallower lesions, a hybrid approach of shallow vHPSD lesions at the posterior aspect and HPSD-AI-guided deeper lesions at the anterior aspect has been suggested. Several studies achieved excellent results in terms of safety, efficacy as well as outcome for this hybrid approach. The POWER PLUS trial was a multicentre, randomized controlled trial that compared procedural efficiency, efficacy, and safety of PVI using vHPSD to standard 35/50 W ablation. Here, vHPSD results in a significant but only modest reduction in procedure time with similar safety and 6-month efficacy in comparison to the standard approach. When using a vHPSD-only protocol, the procedure time (median 70 min, IQR 60–80) was shorter than in the peQasus study. This difference might be attributed to the peQasus study’s inclusion of all comers, while the POWER PLUS study was a multicentre, randomized controlled trial.^25^

Some authors recently adapted the CLOSE-protocol to an individualized and tighter ‘very CLOSE-protocol’ in which an ILD of 3–4 mm at anterior aspect and ILD of 5–6 mm at the posterior aspect of the LA using vHPSD only is performed and safety, efficacy, and follow-up in comparison to conventional CF sensing AI-guided RF ablation has been shown in several studies.^7,11,12,26,27^ PeQasus showed that both approaches seem to create safe and effective PVI with some advantages in terms of short procedures times for the vHPSD approach. Therefore, the choice should be taken by the operator based on the individual workflow and experience.

With comparable efficacy for PVI compared to single-shot-based PVI, the ability to set further ablation strategies as well as an excellent safety profile, the QDOT Micro ablation catheter has the potential for an optimized and adaptable ablation tool. The system, including the catheter and the ablation modes, provides operators with the flexibility to effectively manage and address a wide range of complex arrhythmias.^28,29^ Beside PVI, several case series and case reports are available suggesting further ablation strategies and targets for catheter ablation (atrial tachycardia, CTI block, premature ventricular contractions, and even accessory pathways).^28–34^

The enhanced flexibility of focal RF as compared to single-shot devices is a distinct advantage, as evidenced by the fact that almost one in four patients in the current study received successful extra-pulmonary vein ablation. Furthermore, the ability to toggle between 90 W/4 s and 50 W ablation modalities with the QDOT catheter allows operators to better leverage the different lesion characteristics to the underlying substrate. This was demonstrated in our study too where the majority of ablation on the CTI was performed with the QMODE, and the overwhelming majority on the thin-walled posterior wall in the QMODE+.

The 12-month follow-up was promising and comparable to recent findings of single-shot devices as well as single-tip point-by-point ablation catheters.^2,22–24^ Additionally, no differences were observed when comparing patients of the vHPSD group and hybrid group.

For long-term efficacy, PVI durability is crucial. Data on PVI durability for the cryoballoon showed 56–69% durable isolated PVs while all four PVs were shown to be isolated in 21–26% of patients.^35,36^ Utilizing point-by-point RF ablation via the CLOSE protocol Pooter *et al*. recently showed PVI durability of all PVs in 62% of patients.^37^ Additionally, prior studies reporting on cryoablation or conventional RF showed durability percentages ranging from 0% to 33%.^38^ Latest observations for QDOT-based PVI also observed a high efficacy of the QMODE+ only strategy.^26^ The ‘HPSD Remap’ study compared the QMODE only vs. QMODE+ only PVI procedures’ durability, and a similarly high rate of isolated PVs was found at the 3-month invasive remap.^9^ The durability of PVI with vHPSD-only was high in a real-world registry (81% durable isolated PVs), translating in a favourable long-term clinical success.^14^

### Limitations

The peQasus study is the largest registry on the QDOT Micro ablation catheter to date with the explicit aim to collect large real-world data on this novel catheter ablation technology. Yet, there are some limitations. First, the comparison between the two ablation strategies was not performed by a randomized analysis resulting in potential bias. Indeed, the baseline characteristics of the two groups were slightly different in terms of gender, the presence of hypertension, and congestive heart failure.

Secondly, the follow-up strategy was a pragmatic clinic based one, with a relatively short follow-up period. Thirdly, we did not collect individual lesion level data that may have allowed sophisticated analyses of acute gaps and first-pass isolation rates. Fourthly, due to the retrospective analysis, no data are available on the type of ablation method (HPSD vs. vHPSD) implemented to eliminate remaining gaps in patients without first-pass isolation. Fifthly, a significant difference was found in the utilization of general anaesthesia with a significantly higher rate in the hybrid group. Sixthly, with >20%, a relatively high rate of lost to follow-up was observed, however, there was no difference between the two groups.

### Conclusions

In this extensive multicentre study, the use of temperature-controlled HPSD and vHPSD ablation has demonstrated safe and effective PVI with a relatively short procedure duration. The 12-month follow-up outcomes are encouraging. Notably, no differences were observed in safety and outcomes between the vHPSD-only approach and the hybrid approach.

## Supplementary Material

euae284_Supplementary_Data

## Data Availability

The data will not be available for other researchers due to ethical reasons.

## Appendix 1

### Participating centres of the peQasus study

1. University Heart Center Lübeck, University Hospital Schleswig-Holstein, Department of Rhythmology, Germany
2. Herz- und Diabeteszentrum NRW, Bad Oeynhausen, Germany
3. Heart Rhythm Management Centre, Postgraduate Program in Cardiac Electrophysiology and Pacing, Universitair Ziekenhuis Brussel—Vrije Universiteit Brussel, Belgium
4. Universität Graz, Department of Cardiology, Austria
5. Heart and Vascular Center, Semmelweis University, Budapest, Hungary
6. Department of Internal Medicine and Cardiology University Clinical Center, Medical University of Warsaw, Warsaw, Poland
7. Department of Cardiology, AZ Sint-Jan Hospital, Bruges, Belgium
8. Liverpool Heart and Chest Hospital, Faculty of Health and Life Sciences, University of Liverpool, UK
9. Maastricht University Medical Center, The Netherlands
10. University Heart Center Kiel, University Hospital Schleswig-Holstein, Germany
11. University Hospital of Patras, Greece
12. University Hospital of Besançon, France
13. Oxford University Hospitals NHS Foundation Trust, Oxford, UK
14. Ordensklinikum Linz Elisabethinen, Austria
15. Division of Clinical Electrophysiology, Department of Cardiology, Centre of Postgraduate Medical Education, Grochowski Hospital, Warsaw, Poland.
