# Supplementary material for: Temperature-guided high and very high-power short duration ablation for atrial fibrillation treatment: the peQasus multicentre study
Source: Europace. 2024 Nov 7;27(6):euae284. doi: 10.1093/europace/euae284 (PMC12187331; doi:10.1093/europace/euae284)
Supplement: euae284_Supplementary_Data [file euae284_supplementary_data.pdf]

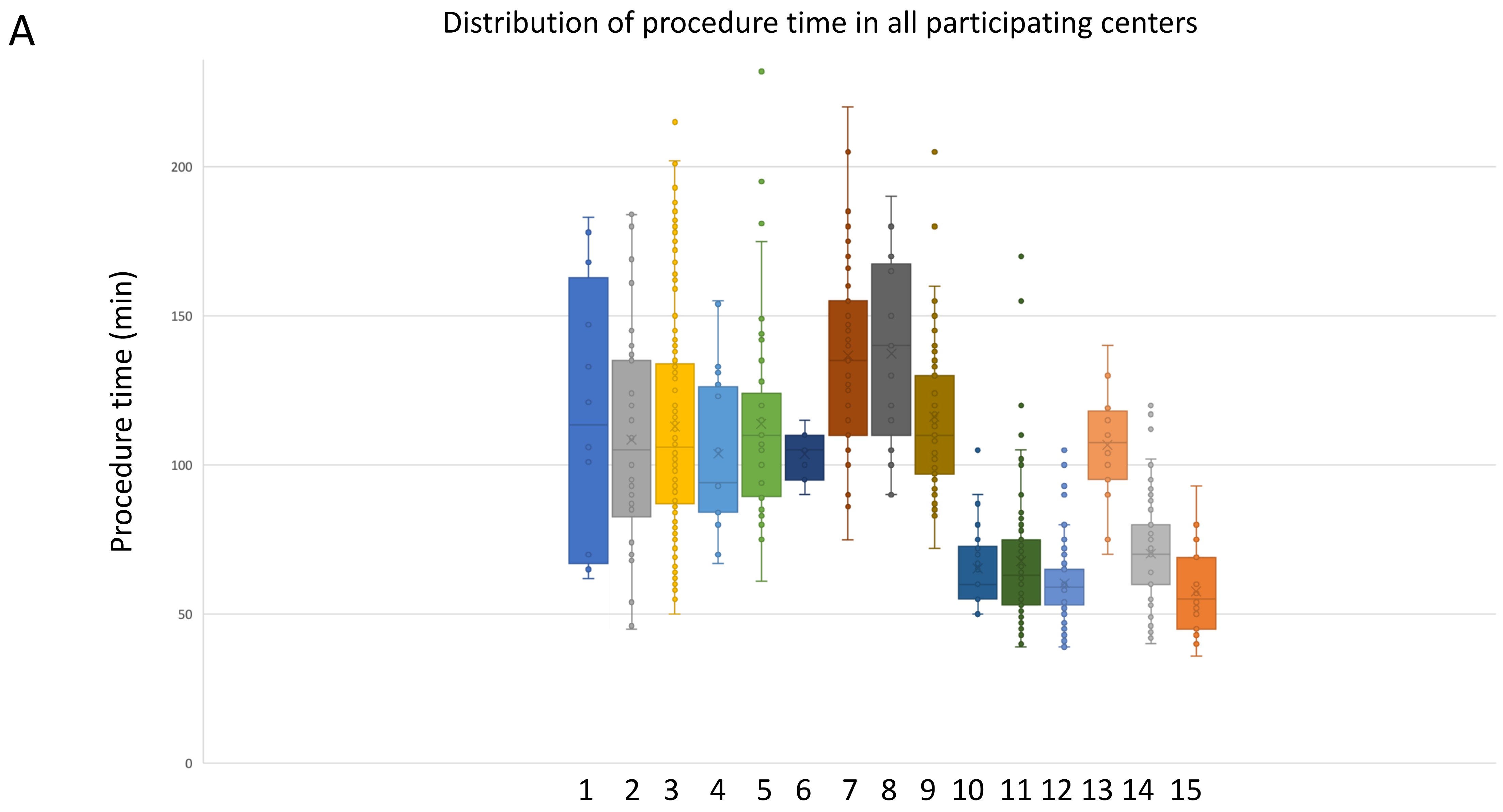
A
Distribution of procedure time in all participating centers
Procedure time (min)
0
50
100
150
200
1
2
3
4
5
6
7
8
9
10
11
12
13
14
15

B

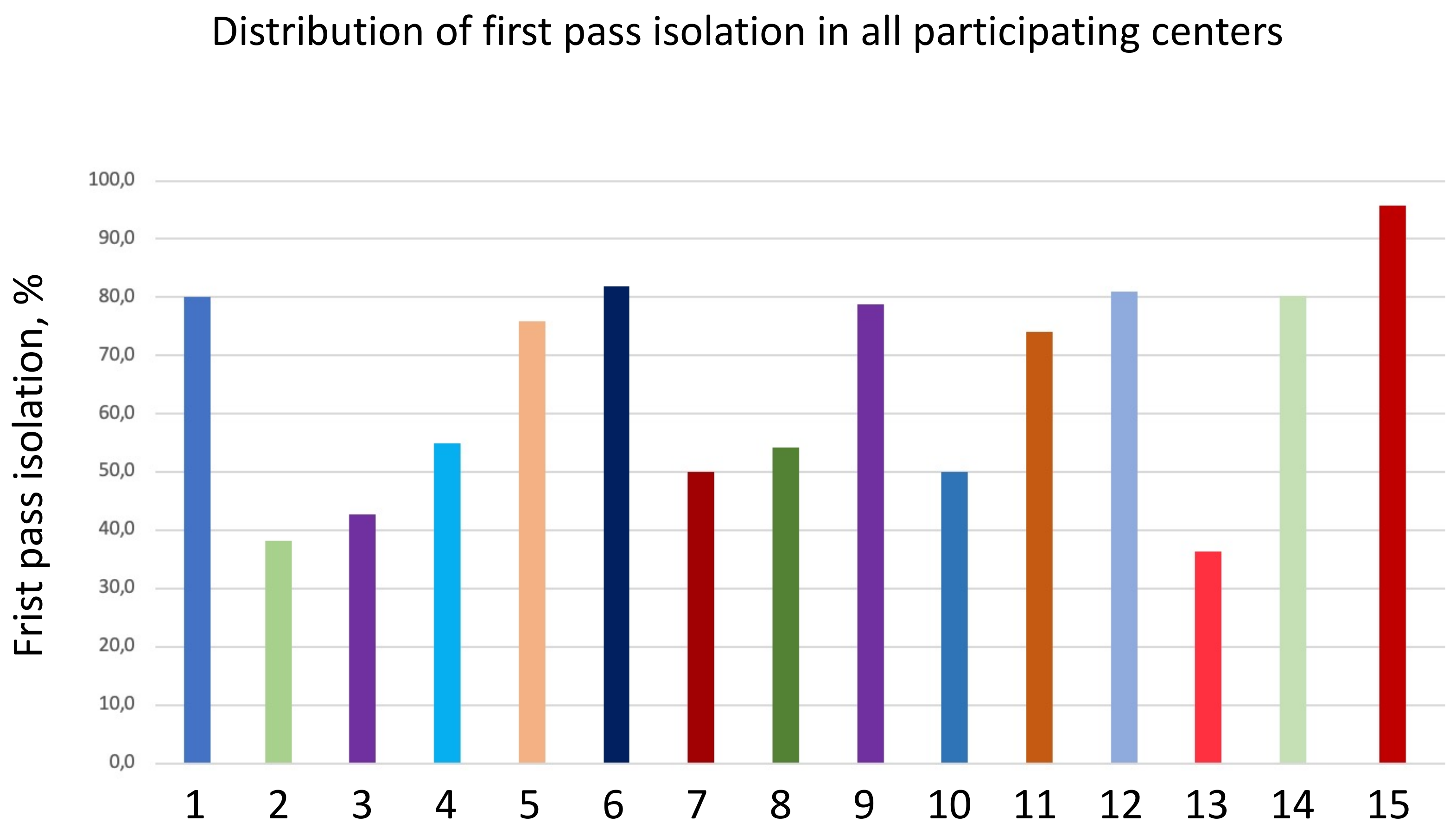
Distribution of first pass isolation in all participating centers
Frist pass isolation, %
100,0
90,0
80,0
70,0
60,0
50,0
40,0
30,0
20,0
10,0
0,0
1
2
3
4
5
6
7
8
9
10
11
12
13
14
15
